# Changes in Depressive Symptoms among Older Adults with Multiple Chronic Conditions: Role of Positive and Negative Social Support

**DOI:** 10.3390/ijerph14010016

**Published:** 2016-12-26

**Authors:** SangNam Ahn, Seonghoon Kim, Hongmei Zhang

**Affiliations:** 1 Division of Health Systems Management and Policy, School of Public Health, The University of Memphis, Memphis, TN 38152, USA; 2 Department of Health Promotion and Community Health Sciences, School of Public Health, Texas A&M University, College Station, TX 77843, USA; 3 School of Economics, Singapore Management University, Singapore 178903, Singapore; seonghoonkim@smu.edu.sg; 4 Division of Epidemiology, Biostatistics, and Environmental Health, School of Public Health, The University of Memphis, Memphis, TN 38152, USA; hzhang6@memphis.edu

**Keywords:** chronic illness, depression, social support, successful aging

## Abstract

Depression severely affects older adults in the United States. As part of the social environment, significant social support was suggested to ameliorate depression among older adults. We investigate how varying forms of social support moderate depressive symptomatology among older adults with multiple chronic conditions (MCC). Data were analyzed using a sample of 11,400 adults, aged 65 years or older, from the 2006–2012 Health and Retirement Study. The current study investigated the moderating effects of positive or negative social support from spouse, children, other family, and friends on the association between MCC and depression. A linear mixed model with repeated measures was used to estimate the effect of MCC on depression and its interactions with positive and negative social support in explaining depression among older adults. Varying forms of social support played different moderating roles in depressive symptomatology among older adults with MCC. Positive spousal support significantly weakened the deleterious effect of MCC on depression. Conversely, all negative social support from spouse, children, other family, and friends significantly strengthened the deleterious effect of MCC on depression. Minimizing negative social support and maximizing positive spousal support can reduce depression caused by MCC and lead to successful aging among older adults.

## 1. Introduction

Depression significantly affects adults aged 65 years and older in the U.S. [1]. Up to 5% of older adults reported having major depression; the prevalence rises to 11.5% among older adults who are hospitalized and 13.5% when older adults require home healthcare [2]. In addition, lifetime experience of depressive disorder among older adults is projected to increase by 117% from 2005 to 2050 [3]. Notably, depressive symptoms can significantly increase morbidity and mortality [4], impair executive function [5], increase disability [6], and erode quality of life [7] among older adults. As the elderly population increases from 40.2 million in 2010 to 88.5 million in 2050 [8], the increasing pattern of late-life depression will escalate pressure on the U.S. healthcare delivery and financing system [1,9,10,11,12].

Recent studies have highlighted the association between multiple chronic conditions and late-life depression [6,13,14]. Although any chronic conditions can produce depressive symptoms, there are certain health conditions strongly associated with depression [6]. Inflammation can cause depression [6,15,16] in the form of obesity [17,18], osteoporosis [19,20], asthma [21,22], and osteoarthritis [23]. Impaired glucose metabolism can also be a risk factor for depression [6,24,25]. Prior studies demonstrated that diabetes elevated the risks of major depression by 15% and depressive symptoms by 20% [26]. Heart diseases can also cause depression due to poor adherence to treatment and lifestyle recommendations; dysfunction in the sympathetic, neuroendocrine, autonomic, immune, and inflammatory systems [27]. In addition, depression is frequently observed among patients with stroke [28], cancer [29], and hypertension [30,31].

Late-life depression, however, can be improved by cultivating the social environment including the groups to which the elderly belong and the neighborhoods in which they live [32]. Social support reflects one of many dimensions of social environment [33] and can affect mental health outcomes among older adults [34,35]. According to the stress-buffering model, positive social support can buffer the deleterious effects of stressful life events (e.g., bereavement, crime, layoff, social network crises) on depressive symptoms among older adults [34,36]. This buffer theory was still applicable when chronic diseases (e.g., arthritis) were positively associated with depressive symptoms, which were mitigated by various aspects of social support (including the presence of partner, having many close social relationships, feelings of mastery, a high self-esteem), especially when persons suffered from severe arthritis [37]. On the other hand, deficits in decent social support can be risk factors for depression in late life [6]. Negative social supports (or social restraints), including marital conflict, perceived family criticism, and depression in the spouse, can predispose older adults to developing or worsening depressive symptomatology and decrease life satisfaction [6,38,39]. Social restraints can be compounded when caregivers are burned out while providing care to their older family members especially those who suffer from multiple chronic conditions (MCC) [40]. Under the circumstances, it is hard to expect these informal caregivers to provide quality care to their care recipients [41].

Nevertheless, the literature is relatively sparse on how positive and negative social support can moderate the negative effects of MCC on depressive symptoms among older adults. Thus, the aims of this study were to (1) describe study participants’ characteristics in terms of depression, number of chronic conditions, and positive/negative social support and (2) identify the moderating effects of positive/negative social support on the association between MCC and depression. The study findings can help policy makers further develop healthy social support systems to reduce depression among older adults with MCC.

## 2. Material and Methods

### 2.1. Sample and Design

The data for the present study come from the Health and Retirement Study (HRS), which is an ongoing, biannual, longitudinal panel study surveying a representative sample of 22,000 Americans over the age of 50 [42]. The present study is limited to participants who responded to the Leave-Behind Questionnaire pertaining to social support and are aged 65 or older. As such, the present study includes HRS interviews conducted in 2006 (*n* = 11,400), 2008 (*n* = 11,349), 2010 (*n* = 10,950), and 2012 (*n* = 10,747), spanning as many as 7 years. Respondents received a self-reported psychosocial questionnaire (including social support variables) every other wave (every 4 years) [43]. At baseline (year of 2006) of the present study, the study sample consisted of individuals age 65–85 years (M = 74.9, SD = 7.6) where 57.3% were female and 68.4% had at least a high school diploma. Most study participants (83.5%) were white while 13.3% were African American. Further details of the HRS design, sampling procedures, data collection, and response rates were reported at a previous study [44]. The study sample size was 11,400 adults aged 65 years or older who reported perceived social support.

### 2.2. Measures

Health conditions of respondents were represented by the number of chronic conditions, commonly associated with depressive symptomatology, including hypertension, diabetes, cancer, chronic lung disease, heart condition, arthritis, stroke, and psychiatric/emotional problems [45]. These conditions were summed up to a score and recoded as 0–6 or higher based on distribution patterns.

The primary dependent variable in the study was depressive symptomatology, which was measured by an index summarized from eight symptoms of depression (felt depressed, everything was an effort, restless sleep, was (not) happy, felt lonely, (did not) enjoy life, felt sad, could not get going), ranging from 0 to 8 [46]. These items were based on the Center for Epidemiological Studies-Depression (CES-D) scale [47]. This scale was reported to have high internal consistency, reliability [48] and has been used for assessing the severity of depressive symptoms among older adults [46,49,50,51]. Respondents were asked how frequently they had experienced each symptom during the past week: 1 (“all or almost all”), 2 (“most of the time”), 3 (“some of the time”), and 4 (“none or almost none”). These responses were converted to Yes/No by setting “all” and “most” of the time equal to “Yes” and “some” and “none” of the time equal to “No”. Created by summing the number of “Yes” answers across the eight items, a higher score (ranging from zero to eight) indicates greater depression symptomatology.

Positive and negative social supports were composed based upon a series of questions assessing quality of social ties and the quality of interaction with those social ties [52]. Of 4 relationship categories (i.e., spouse, children, other family, friends), there are 3 positively worded items (e.g., “how much do they really understand the way you feel about things?”) and 4 negatively worded items (e.g., “how often do they make too many demands on you?”) with 4 possible answers: 1 (“a lot”), 2 (“some”), 3 (“a little”), and 4 (“not at all”). After reversing these responses and averaging the scores within each dimension, a higher value indicates higher positive or negative social support. A previous report revealed psychometrics of alpha reliability across 4 relationship categories (positive social support from spouse = 0.81; positive social support from children = 0.83; positive social support from other family = 0.86; positive social support from friends = 0.84; negative social support from spouse = 0.78; negative social support from children = 0.78; negative social support from other family = 0.78; negative social support from friends = 0.76) [53].

Covariates included a set of demographic factors such as age, education (less than high school, GED, high school graduate, some college, college and above), sex, and race/ethnicity (white, black/African American, other).

### 2.3. Data Analysis

A linear mixed model with repeated measures for survey data using Stata 14.0 (StataCorp LP, College Station, TX, USA) was used to estimate the effect of MCC and its interactions with positive and negative social support factors in explaining depression among older adults (i.e., eight independent models). In these models, we controlled for age, gender, educational attainment, race/ethnicity, and survey wave.

In order to account for the panel nature of the data, we used the person-level weight provided by the HRS, which makes our sample a national representation of the non-institutionalized population in the U.S. aged 65 and older. Since the dependent variable and covariates were measured several times over different survey waves, we clustered standard errors at the person level to adjust for potential correlation of errors over time within the same person.

## 3. Results

Study participants’ depression symptoms tended to improve between 2006 and 2010 but they slightly worsened in 2012 (*p* < 0.001) (Table 1). During this period, study participants reported having an increasing number of chronic conditions from 2.37 in 2006, 2.48 in 2008, 2.59 in 2010, and to 2.71 in 2012. However, we did not find statistically significant mean differences between the 2006 wave and the 2012 wave in terms of any positive or negative social support.

**Figure 1:**
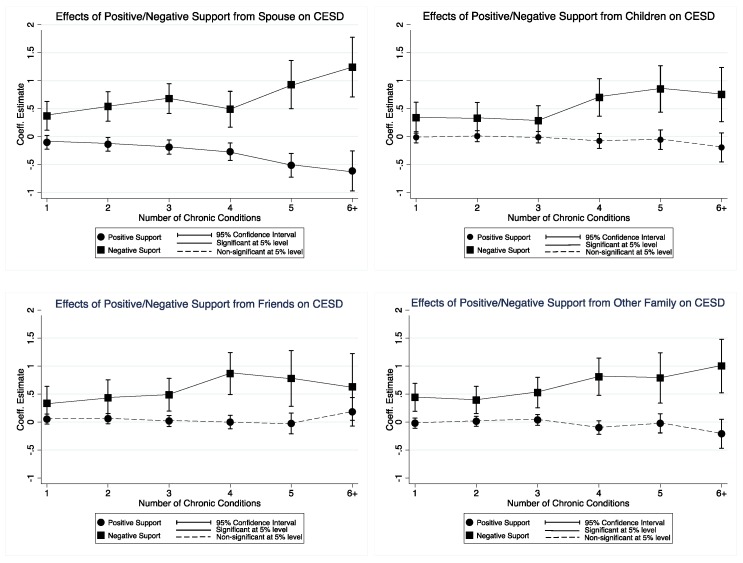
Effects of positive/negative supports from spouse, children, other family, and friends on CESD depression.

Results of a series of linear mixed models (i.e., eight independent models) showed main and interaction effects of positive/negative social support on the association between MCC and depression (Table 2). Only positive spousal support (Model 1) significantly weakened the deleterious effect of MCC on depression (*p* < 0.001). By contrast, all negative social support from spouse (Model 2), children (Model 4), other family (Model 6), and friends (Model 8) significantly strengthened the deleterious effect of MCC on depression (all *p*-values < 0.001). We depicted Figure 1 to further visualize the interaction effects of positive/negative social support on the associations between MCC and depression, suggesting a moderating effect. Although not all interactions were statistically significant, a clear trend was observed in Figure 1. That is, level of depression tends not to be influenced by the number of chronic conditions when receiving positive social support (except positive spousal support), and increased with increasing chronic conditions when receiving negative social support.

## 4. Discussion

As a stress-buffering model suggests [54,55], we examined interaction effects of social support on late-life depression when study participants have multiple chronic conditions. To our best knowledge, it is the first effort to investigate the moderating effect of varying forms of social support as part of the social environment on the association between MCC and depression symptoms among older adults. While previous studies found significant effects of social support on mental health [56,57], the current study specifically suggested how varying social support plays different moderating roles in depressive symptomatology among older adults with MCC. Indeed, positive spousal support weakened the deleterious effect of MCC on depression while negative support from the spouse, children, other family, and friends strengthened this effect. These results suggest the importance of helping adults with MCC minimize potential negative social support while strengthening positive spousal support.

Most of all, the current study confirmed previous studies regarding the positive mental health effect of having positive spousal support for older adults with chronic conditions [58], weakening the negative effect of MCC on depressive symptomatology. A previous study similarly found that a high level of spousal support weakened the detrimental association between disease severity and depression while a low level strengthened this association [59]. It was also found that a husband’s positive support significantly improved a wife’s mental status especially when she had breast cancer [60]. In spite of this positive health aspect of having a spouse, there is an increasing trend of women living without a spouse from 35% in 1950, 49% in 2000, to 51% in 2005 [61]. Living without a spouse (especially bereavement) has a negative mental health effect including grief and depression in late life [54]. In a meta-analysis, spousal care was only successful in reducing patients’ depressive symptoms compared to forms of support from other family members [62]. The current study also found that this positive effect of social support was not shown with support from other family, children, or friends. This is presumably because the spouse often has the greatest potential to affect the patient’s health due to the number of opportunities for support provision and intimate relationship [63]. Quality spousal care, filled with better understanding and affection, can help care recipients cope well with pain and symptoms related to chronic conditions [64], which in turn could improve depression [65]. Nevertheless, more studies should be done to better investigate the underlying mechanisms of the effects of positive spousal care on improving depression.

On the other hand, we also found there was a deleterious effect of negative spousal support on depression among older adults with MCC. A previous study found that spouses’ negative (pressuring) support worsened the health status of older adults with osteoarthritis [64]. Depressive symptomatology of older adults who had MCC can be especially worsened when they reported having negative spousal support [66]. Another study showed that negative interaction with a husband increased depressive symptomatology and decreased self-care behaviors when a wife suffered from osteoarthritis [67]. Even spousal disability increased levels of emotional loneliness [68]. These studies encourage us pay more attention to the quality of spousal care and marriage in later life to help improve patients' mental health [58]. It is easy to speculate that care provision for a spouse with MCC could generate a high level of caregiver burden or fatigue that can be a result of an imbalance between caregiving demands and caregiving resources and, therefore, lead to negative health outcomes among caregivers [69,70]. Compared to non-caregiving control groups, older adult spousal caregivers experienced more cognitive functioning difficulties, stress, loneliness, depression, anxiety, and poorer mental health [71]. On the other hand, spouses with greater network support experienced fewer depressive symptoms when the patient’s illness was more severe [59]. Preparing resources and maintaining strong social support systems could improve the health status of older family caregivers, which helps to ease caregiver fatigue and results in better care for their spouses [72].

Interestingly, the current study found that negative social support from other family members, adult children, and friends strengthened the deleterious effect of MCC on depression among older adults. It is often cited that older adults tend to feel emotional and lonely when they have smaller social networks and less contact with children [58]. However, the current study was not able to detect any positive effect of social support from other family, children, and friends. Instead, the current study indicated that troubled family or friends’ relationships could have a harmful effect on mental health among older adults with MCC. As a reminder, HRS asked older adults experiencing negative social support about the extent of them: (1) making too many demands on you; (2) criticizing you; (3) letting you down when you are counting on them; and (4) getting on your nerves. Although it is beyond the scope of this study, the negative health effects of support from friends or family members found in this study point to potential elder abuse, neglect, or exploitation that may be prevalent (but under-reported) and committed by older adults’ friends or family members [73]. A meta-analysis suggested that risk factors of elder abuse include the perpetrator (caregiver burden or stress, psychiatric illness or psychological problems), relationship (family disharmony, poor or conflictual relationships), and environment (low social support, living with others) [73]. While referring to a high prevalence of elder abuse (10% in the U.S.) in community-dwelling older adults [74], older adults with MCC can be susceptible targets for perpetrators because they are prone to being disabled and having cognitive impairment [75,76]. Nevertheless, the study findings do not negate the importance of social support from others; instead, the current study warns of potential negative mental health impacts of bad or low quality relationships, especially when older adults carry the heavy burden of chronic conditions. Closely working with Adult Protective Services [77], primary care physicians should be more attentive to any irregular symptoms of elder abuse or negligence when the depression of elderly patients with MCC worsens.

Several limitations of the current study should be noted. First, the current study is based on an observational study, which did not allow for causal examination. Second, in spite of having valid psychometrics, all measures are self-reported (including MCC, CES-D depression, social supports), introducing potential reporting bias relative to more clinically based data [78]. Third and last, this study was limited in its measure of in-depth negative and positive social support mechanisms including underlying reasons (especially for potential elder abuse or negligence as part of negative social supports). Future studies need to construct more valid models to attain a better understanding of the dynamics of MCC, depression, and social support among older adults.

## 5. Conclusions

We believe this study makes an important contribution to the gerontological literature by showing the moderating effects of varying forms of social support on the association between MCC and depression among older adults. The current study confirmed the importance of quality spousal support and care as part of a positive social environment to maintain the mental health of older adults when they have MCC. At the same time, our findings suggest that identifying and minimizing the deleterious health effect of negative social supports could help older adults improve their depression, which could in turn help them manage their chronic conditions and experience positive aging. At the community-level, concerted efforts are needed to first identify at-risk older adults with MCC, assure their social connectedness with family members and local communities, and closely monitor their mental health status related to MCC.

## Figures and Tables

**Table 1:** Description of variables of interest by wave.

Variables	2006 (*n* = 11,400)	2008 (*n* = 11,349)	2010 (*n* = 10,950)	2012 (*n* = 10,747)	*p*-Value
	Mean (SD)				
CESD depression (0–8)	1.48 (1.93)	1.41 (1.91)	1.34 (1.86)	1.39 (1.89)	<0.001
Number of chronic conditions (0–6)	2.37 (1.42)	2.48 (1.44)	2.59 (1.43)	2.71 (1.45)	<0.001
Positive social support					
From spouse (1–4)	2.94 (1.01)	2.95 (1.02)	3.02 (0.99)	2.97 (1.00)	0.2147
From children (1–4)	2.70 (1.04)	2.71 (1.05)	2.68 (1.04)	2.69 (1.04)	0.7066
From other family (1–4)	2.23 (1.04)	2.26 (1.04)	2.22 (1.05)	2.25 (1.04)	0.3899
From friends (1–4)	2.21 (1.05)	2.24 (1.06)	2.21 (1.04)	2.25 (1.06)	0.0784
Negative social support					
From spouse (1–4)	1.55 (0.48)	1.55 (0.50)	1.54 (0.48)	1.54 (0.48)	0.5833
From children (1–4)	1.41 (0.42)	1.39 (0.42)	1.40 (0.43)	1.39 (0.43)	0.1192
From other family (1–4)	1.33 (0.41)	1.32 (0.42)	1.32 (0.42)	1.32 (0.43)	0.2399
From friends (1–4)	1.27 (0.34)	1.25 (0.32)	1.26 (0.32)	1.26 (0.34)	0.1206

Notes: SD: standard deviation; CESD: Center for Epidemiologic Studies Depression; *p*-values are based on the *t*-statistics of the group mean difference tests between the first (2006) and last (2012) waves.

**Table 2:** Adjusted moderating effect of social support on the association between Multiple Chronic Conditions (MCC) and CESD depression.

CESD Depression		Positive Support				Negative Support				
		Coef.	*p*-Value	95% CI			Coef.	*p*-Value	95% CI	
Model 1		Spouse				Model 2	Spouse			
	MCC	0.489	<0.001	0.387	0.591		0.033	0.489	−0.060	0.125
	Support from spouse	−0.110	0.005	−0.186	−0.033		0.115	0.152	−0.042	0.273
	MCC × Support from spouse	−0.071	<0.001	−0.103	−0.039		0.155	<0.001	0.098	0.212
	Intercept	0.912	0.001	0.398	1.426		0.142	0.575	−0.353	0.636
	R-squared	0.1513					0.1474			
Model 3		Children				Model 4	Children			
	MCC	0.367	<0.001	0.294	0.439		0.112	0.007	0.031	0.193
	Support from children	−0.148	<0.001	−0.205	−0.090		0.162	0.042	0.006	0.318
	MCC × Support from children	−0.017	0.166	−0.040	0.007		0.141	<0.001	0.086	0.196
	Intercept	0.218	0.284	−0.181	0.618		−0.324	0.156	−0.772	0.124
	R-squared	0.1306					0.1374			
Model 5		Other family				Model 6	Other family			
	MCC	0.340	<0.001	0.280	0.400		0.084	0.034	0.006	0.161
	Support from other family	−0.054	0.050	−0.109	0.000		0.091	0.231	−0.058	0.240
	MCC × Support from other family	−0.018	0.118	−0.041	0.005		0.163	<0.001	0.107	0.219
	Intercept	0.330	0.097	−0.059	0.719		−0.057	0.797	−0.488	0.375
	R-squared	0.1196					0.1341			
Model 7		Friend				Model 8	Friend			
	MCC	0.298	<0.001	0.241	0.354		0.111	0.01	0.027	0.196
	Support from friend	−0.047	0.09	−0.101	0.007		0.049	0.601	−0.135	0.234
	MCC × Support from friend	0.004	0.713	−0.019	0.027		0.151	<0.001	0.087	0.216
	Intercept	0.231	0.253	−0.165	0.628		−0.091	0.682	−0.530	0.347
	R-squared	0.1133					0.1208			

Notes: All models (Model 1–8) were independently conducted to investigate the interaction effects between varying types of social support and MCC on CESD depression. We control for age, education, gender, race/ethnicity, and study year (wave) in all models. Standard errors are clustered at the person level. CESD: Center for Epidemiologic Studies Depression. MCC: multiple chronic conditions.
